# Enterohepatic *Helicobacter* in Ulcerative Colitis: Potential Pathogenic Entities?

**DOI:** 10.1371/journal.pone.0017184

**Published:** 2011-02-23

**Authors:** John M. Thomson, Richard Hansen, Susan H. Berry, Mairi E. Hope, Graeme I. Murray, Indrani Mukhopadhya, Mairi H. McLean, Zeli Shen, James G. Fox, Emad El-Omar, Georgina L. Hold

**Affiliations:** 1 Gastrointestinal Research Group, Division of Applied Medicine, University of Aberdeen, Foresterhill, Aberdeen, United Kingdom; 2 Child Health, University of Aberdeen, Royal Aberdeen Children's Hospital, Foresterhill, Aberdeen, United Kingdom; 3 Department of Pathology, University of Aberdeen, Foresterhill, Aberdeen, United Kingdom; 4 Division of Comparative Medicine, Massachusetts Institute of Technology, Cambridge, Massachusetts, United States of America; Queen Mary University of London, United Kingdom

## Abstract

**Background:**

Changes in bacterial populations termed “dysbiosis” are thought central to ulcerative colitis (UC) pathogenesis. In particular, the possibility that novel *Helicobacter* organisms play a role in human UC has been debated but not comprehensively investigated. The aim of this study was to develop a molecular approach to investigate the presence of *Helicobacter* organisms in adults with and without UC.

**Methodology/Principal Findings:**

A dual molecular approach to detect *Helicobacter* was developed. Oligonucleotide probes against the genus *Helicobacter* were designed and optimised alongside a validation of published *H. pylori* probes. A comprehensive evaluation of *Helicobacter* genus and *H. pylori* PCR primers was also undertaken. The combined approach was then assessed in a range of gastrointestinal samples prior to assessment of a UC cohort. Archival colonic samples were available from 106 individuals for FISH analysis (57 with UC and 49 non-IBD controls). A further 118 individuals were collected prospectively for dual FISH and PCR analysis (86 UC and 32 non-IBD controls). An additional 27 non-IBD controls were available for PCR analysis. All *Helicobacter* PCR-positive samples were sequenced. The association between *Helicobacter* and each study group was statistically analysed using the Pearson Chi Squared 2 tailed test. *Helicobacter* genus PCR positivity was significantly higher in UC than controls (32 of 77 versus 11 of 59, p = 0.004). Sequence analysis indicated enterohepatic *Helicobacter* species prevalence was significantly higher in the UC group compared to the control group (30 of 77 versus 2 of 59, p<0.0001). PCR and FISH results were concordant in 74 (67.9%) of subjects. The majority of discordant results were attributable to a higher positivity rate with FISH than PCR.

**Conclusions/Significance:**

*Helicobacter* organisms warrant consideration as potential pathogenic entities in UC. Isolation of these organisms from colonic tissue is needed to enable interrogation of pathogenicity against established criteria.

## Introduction

Ulcerative colitis (UC) is a chronic condition of the human colon which affects the superficial mucosal layer from the rectum and extending proximally for variable distances [1]. This variable phenotype remains a puzzle, as does our difficulty in achieving long-term cure with current treatments. Recent developments in genetics have greatly improved our understanding of the inflammatory bowel diseases (Crohn's disease and UC), resulting in a renewed interest in the interplay between host immunology and bacteria at the mucosal surface; however genetic elements appear to be more important in Crohn's disease (CD) than UC. The possibility of infection as a trigger event for, or indeed as the cause of, inflammatory bowel disease (IBD) has long been debated with various organisms being suggested as pathogens. None of these organisms have been conclusively proven as causative agents. Studies examining the diversity of bacteria in IBD have shown increased cell counts of bacteria and reduced bacterial diversity. Changes in bacterial populations to the detriment of the host have been termed “dysbiosis” and this change is thought central to IBD pathogenesis. IBD onset following infectious episodes is well described and one possibility is that gastrointestinal infection may facilitate dysbiosis and ultimately IBD. Whether acute self-limiting infection is sufficient as a single entity, or whether chronic infection with as yet unknown agents is required to drive the chronicity of disease is unknown. UC is a stronger candidate than CD for a purely infectious aetiology because of the weaker genetic association, continuity of disease distribution and the relative limitation of disease to superficial tissue. It is likely however that a combination of host (genetic) susceptibility, a trigger event (which may be infectious) and the progression to dysbiosis are all likely required for the development of IBD.

The discovery that *Helicobacter pylori* was the causative agent underpinning gastric and duodenal ulceration and ultimately gastric cancer revolutionised our understanding of these conditions and resulted in a Nobel prize for Robin Warren and Barry Marshall. The tantalising possibility that a similar agent is responsible for IBD warrants consideration and exploration [2].

The family *Helicobacteraceae* contains the genera *Helicobacter* and *Wolinella*. The *Helicobacter* genus can be split into two groups, gastric *Helicobacter*, describing those that preferentially colonise the stomach, and enterohepatic *Helicobacter*, which preferentially colonise the intestinal or hepatobilliary system (Table 1). Enterohepatic *Helicobacter* organisms have been cultured from both Cotton-top tamarin monkeys (*Saguinus oedipus*) and rhesus monkeys (*Macaca mulatta*) with colitic disease (*Helicobacter* sp. Flexispira taxon 10, *Helicobacter macacae* and *Helicobacter* sp. Rhesus monkey 2), whilst *Helicobacter hepaticus* and *Helicobacter bilis* have been shown to be capable of causing IBD-like disease in immunodeficient rodent models [3]–[5]. Thus animal models demonstrating that infection with *Helicobacter* spp. on a background of host immunodeficiency can lead to colitis, and that “auto-immune” type reactions to commensal bacteria can be initiated by such organisms, would suggest the possibility of parallel mechanisms in humans resulting in IBD.

**Table 1:** Classification of named *Helicobacter* spp. as Gastric or Enterohepatic.

Gastric	Enterohepatic
*H. acinonychis*	*H. anseris*
*H. aurati*	* *H. bilis*
*H. bizzozeroni*	*H. brantae*
*H. cetoreum*	* *H. canis*
*H. felis*	* *H. canadensis*
*H. mustelae*	*H. cholecystus*
* *H. pylori*	* *H. cinaedi*
*H. salomonis*	*H. equorum*
*H. suis*	* *H. fennelliae*
*H. bovis* (candidate species)	*H. ganmani*
*H. suncus* (candidate species)	*H. hepaticus*
*H. cyanogastricus*	*H. mastomyrinus*
	*H. marmotae*
	*H. mesocricetorum*
	*H. magdeburgensis*
	*H. muridarum*
	*H. pametenis*
	* *H. pullorum*
	*H. rodentium*
	*H. suncus*
	*H. trogontum*
	*H. typhlonicus*
	*H. westmeadii*
	* *H. winghamensis*

*Isolated from humans.

Various groups have examined human IBD for the presence of *Helicobacter* spp., from the negative studies of Bell and Grehan [6], [7], through to studies by Bohr, Zhang and Laharie which have successfully demonstrated PCR evidence of non-*pylori Helicobacter* (n*pH*) in both IBD and controls [8]–[10]. The methodologies used, the variable rates of positivity reported between groups, and the small study numbers included in some, mean discussions at *Helicobacter* species level have been limited. Unfortunately, no-one has successfully cultured non-*pylori Helicobacter* organisms from IBD tissue (although 7 enterohepatic *Helicobacter* spp. have been cultured from the gastrointestinal tract of humans with diarrhoea or systemic disease [2]). The difficulties in isolating and culturing non-*pylori Helicobacter* from human colonic tissue highlight the importance of molecular approaches as viable alternatives to facilitate the study of the role of *Helicobacter* spp. in extra-gastric diseases. However, it is vital that these molecular methods are suitably sensitive, specific and applicable to a diverse range of samples. The purpose of the present study was to design a combined molecular approach to identify *Helicobacteraceae* organisms within a variety of gastrointestinal sample types. Our specific aim was to examine colonic tissue from IBD patients to assess the prevalence of *Helicobacteraceae* organisms against tissue from controls largely undergoing colorectal cancer screening. We elected to analyse UC cases rather than CD cases for the reasons outlined above regarding UC as a stronger candidate for an infectious aetiology.

## Methods

### Ethics Statement

Ethical approval for the archival specimen analysis and the biopsy study was granted by North of Scotland Research Ethics Service and written informed consent was obtained from all subjects in the biopsy study.

### Development of a Combined Molecular Approach for the Detection of *Helicobacteraceae* Organisms

#### Development of PCR Methodology

A bacterial reference panel was used to screen a series of primer combinations. The bacterial strains used in this study included: *Helicobacter bilis* (ATCC 51630), *H. canadensis* (ATCC 700968), *H. canis* (ATCC 51402), *H. cholecystus* (ATCC 700242), *H. cinaedi* (CCUG 18818), *H. felis* (ATCC 49179), *H. hepaticus* (ATCC 51449), *H. pullorum* (NCTC 12824), *H. pylori* (ATCC 700392), *Pseudomonas fluorescens* (clinical isolate), *Listeria monocytogenes* (clinical isolate), *Aeromonas caviae* (clinical isolate), *Aeromonas sobria* (clinical isolate), *Campylobacter jejuni* (clinical isolate), *Proteus mirabilis* (NCTC 3177), *Enterobacter aerogenes* (NCIMB 10102), *Yersinia enterocolitica* (NCIMB 2124), *Bifidobacterium longum* (NCIMB 8809), *Bifidobacterium infantis* (DSM 20088), *Eubacterium rectale* (NCIMB 14373), *Roseburia intestinalis* (DSM 14610), *Bacteroides vulgatus* (DSM 1447), *Bacteroides thetaiotaomicron* (NCTC 10582), *Eubacterium hallii* (DSM 17630), *Enterococcus faecalis* (NCIMB 13280), *Pseudomonas aeruginosa* (NCIMB 8626), *Enterobacter cloacae* (NCIMB 8556), *Proteus vulgaris* (NCTC 4175), *Salmonella enteritidis* (NCTC 12694), *Salmonella poona* (NCTC 4840), *Salmonella typhimurium* (NCIMB 13284), *Escherichia coli* (NCIMB 12210), *Shigella sonnei* (ATCC 25931), *Staphylococcus epidermidis* (NCIMB 8853), *Bacillus subtilis* (NCIB 8054), *Bacillus cereus* (ATCC 10876), *Klebsiella pneumoniae* (NCIMB 13281), *Staphylococcus aureus* (NCIMB 12702), *Streptococcus gordonii* (ATCC 35105), *Faecalibacterium prausnitzii* strain A2-165 (DSM 17677), *Megasphaera elsdenii* (ATCC 25940), *Bifidobacterium adolescentis* isolate L2-32, *Lactococcus lactis* strain MG1363, *Enterococcus faecalis* strain JH2-2, *Ruminococcus albus* strain SY3, *Ruminococcus flavefaciens* strain 17, *Eubacterium cylindroides* strain T2-87, *Coprococcus spp* L2-50, *Methanobrevibacter smithii* ATCC 35061, *Acinetobacter baumanii*, *Lactobacillus acidophilus* (ATCC 43561) and *Streptococcus bovis* strain *Z6*. Aerobic and microaerobic strains were grown at 37°C on Columbia agar with 10% horse blood. Anaerobic strains were grown at 37°C on M2GSC [11], MRS or M17 media (Becton Dickinson, Oxford, UK). All strains were used in fluorescent *in-situ* hybrisation (FISH) and PCR optimisation studies.

Initial assessment used a universal 16S bacterial PCR described previously [12]. To allow identification of the family *Helicobacteraceae* (genera *Helicobacter* and *Wolinella*), 8 *Helicobacteraceae* PCR primer pairs were assessed. The nested PCR combination of C05 and C97 [13] followed by a reverse complement of primer C98 [13] and1067r [14] was selected as it yielded a final product of suitable length (∼400 bp) for sequence analysis. For *H. pylori* specific PCR, numerous *H. pylori* specific primer sets targeting the 16S rRNA gene were assessed with the most successful pairing being identified as 27f (5′-AGAGTTTGATCMTGGCTCAG-3′) [15] and HPY (5′-CTGGAGAGACTAAGCCCTCC-3′) [16]. Both PCRs utilised the following conditions: denaturation at 94°C for 5 min, followed by 35 cycles of 94°C for 1 min, 66°C for 1 min, 72°C for 2 min. Final extension 72°C for 10 min. To determine the sensitivity of the genus PCR, decreasing amounts of *H. pylori* and *H. hepaticus*-derived DNA were spiked into faecal samples which were previously analysed by FISH and PCR and found to be negative for *Helicobacteraceae*. Dilutions ranged from 500 pg to 0.05 pg of *Helicobacter* DNA with the detection level of 0.5 pg *Helicobacter* DNA (representing approximately 30 bacteria) consistently being achieved.

#### Development of FISH Methodology

Five broad-specificity probes were designed to target the small subunit rRNA of the family *Helicobacteraceae* (Table 2). The new probes were designed with the Primrose software package [17], checked against the Ribosomal Database Project (RDP) and EMBL databases, and were named according to the nomenclature suggested by the Oligonucleotide Probe Database (OPD) [18]. One of the designed *Helicobacteraceae* probe sequences S-G-Hel-1047-a-A-21 had been previously described as a *Helicobacter* genus specific PCR primer [14] but to our knowledge it has not been used as a probe. Of note, *in-silico* analysis indicated that this probe detects several bacteria of the genera *Sulfurimonas* (17 of 21), *Sulfurovum* (9 of 42) and *Wolinella* (4 of 5). The specificity of the 5 newly designed *Helicobacteraceae* probes along with four previously published *H. pylori* specific probes (Table 2) was tested by whole-cell *in situ* hybridization against a panel of 60 reference strains derived from the human and animal gastrointestinal tract (see above) including a panel of 9 *Helicobacter* type strains [19]. As a positive control for the presence of bacteria, the bacterium-specific probe S-D-Bact-0338-a-A-18 (termed Eub338) was used [20]. Following assessment of the various *Helicobacteraceae* probes, it was identified that the *Helicobacteraceae* probe S-G-Hel-1047-a-A-21 and the *H. pylori* specific probe Hp16S2 hybridized only to the respective target organisms but not to any of the other organisms tested. Both the Eub338 and Hp16S2 hybridised at 50°C and could be co-hybridised using discriminating fluorescent labels (Rhodamine red and Oregon green 488 respectively). S-G-Hel-1047-a-A-21 hybridised at 52°C.

**Table 2:** Probes used for fluorescent *in-situ* hybridisation (FISH).

Probe	16S rDNA position a b	Probe sequence c	Fluorophore	Reference
***Universal***				
Eub A	338–355 a	5′ - GCT GCC TCC CGT AGG AGT - 3′	Rhodamine red	[20]
Eub B	338–355 a	5′ - GCT GCC ACC CGT AGG TGT - 3′	Rhodamine red	[40]
Eub C	338–355 a	5′ - GCA GCC ACC CGT AGG TGT - 3′	Rhodamine red	[40]
***Helicobacteraceae*** ** family specific**				
Hgen1	218–235 b	5′- ARC TGA TAG GAC ATA GRC - 3′ c	Cy3	This study
Hgen2	666–683 b	5′ - TGA GTA TTC YTC TTG ATM - 3′ c	Oregon green 488	This study
Hgen3	657–674 b	5′ - CTC TTG ATC TCT ACG GAT - 3′	Oregon green 488	This study
Hgen4	630–647 b	5′ - ACA CCA AGA ATT CCA CCT - 3′	Oregon green 488	This study
Hgen5	1047–1067 b	5′ - GCC GTG CAG CAC CTG TTT TCA - 3′	Oregon green 488	This study
***Helicobacter pylori*** ** specific**				
Hpy-1	547–567 b	5′- CACACCTGACTGACTATCCCG - 3′	Cy3	[21]
HP2	796–815 b	5′- CTG GAG AGA CTA AGC CCT CC - 3′	Oregon green 488	[16]
Hp16S-1	163–185 b	5′- GGAGTATCTGGTATTAATCATCG - 3′	Oregon green 488	[41]
Hp16S-2	206–227 b	5′- GGACATAGGCTGATCTCTTAGC -3′	Oregon green 488	[41]

a Indicates *E. coli* numbering.

b Indicates *H. pylori* numbering to strain 26695 (ATCC 700392).

c Indicates degeneracy of nucleotides according to IUPAC see http://www.chem.qmul.ac.uk/iubmb/misc/naseq.html.

#### Validation of Molecular Methods (PCR/FISH) for the Detection of *Helicobacteraceae* Organisms

In order to calculate the sensitivity and specificity of the dual molecular approach 100 gastric samples were selected on the basis of *H. pylori* status (50 positive and 50 negative). *H. pylori* status had been confirmed previously by CLO test and histology; however this was blinded to researchers until after molecular assessment. FISH was performed on archival paraffin tissue sections and PCR was performed on DNA extracted from fresh biopsies.

Biopsy blocks were cut to a thickness of 4 µm using a Leica RM2125RT rotary microtome with sections cut per block, and mounted on ChemMate capillary gap slides, 75 µm, (DakoCytomation, Cambridgeshire, UK). Following microtome sectioning and mounting of tissue, slides were dried vertically at room temperature and incubated overnight at 37°C to ensure that the tissue was adhered to the slide. Slides were then arranged by patient and block number and sections 1, 3 and 5 were used for assessing the presence of *H. pylori* coupled with the universal bacterial probe. Sections 2, 4 and 6 were used for assessing the presence of all *Helicobacteraceae*. Biopsy sections were deparaffinised using xylene and ethanol [21]. For glass slides carrying deparaffinised tissue sections, 50 µl of hybridisation buffer was added and coverslips were used to minimise evaporation. Hybridisation was performed for 16 hours for all tissue sections and Vectashield Hardmount (Vector Laboratories, Peterborough, UK) was used.

DNA extraction of mucosal biopsies was performed using the commercially available Qiagen QIAamp Mini kit (Qiagen Crawley UK) with the following amendments. Biopsy samples were kept frozen until the addition of ATL buffer before allowing biopsies to equilibrate to room temperature, an additional 10 µl of Proteinase K was added for an initial lysis period of 18 hours to ensure complete lysis of the biopsy material prior to DNA extraction. PCR was performed as described above with biopsy DNA initially subjected to universal bacterial PCR [12] to confirm the suitability of the DNA for further analysis.

One hundred infectious diarrhoea samples were also collected for inclusion in the validation cohort. Samples were obtained from the Department of Medical Microbiology (Aberdeen Royal Infirmary, Aberdeen) and DNA was extracted using the Nucleon phytopure DNA extraction kit. PCR was performed as described above with faecal DNA initially subjected to universal bacterial PCR [12] to confirm the suitability of the DNA for further analysis.

### Assessment of *Helicobacteraceae* Prevalence in Human Colonic Tissue Using the Combined Molecular Approach

#### Archival colonic tissue specimens

Paraffin embedded colonic specimens from a total of 106 patients were obtained from the Department of Pathology (Aberdeen Royal Infirmary). Fifty-seven ulcerative colitis (UC) patients and forty-nine healthy controls (HC) were included. All UC patients were assessed during active disease and analysis was performed on all available colonic sites that were biopsied at the time of colonoscopy. The HC subjects comprised individuals who had undergone a colonoscopy in which colonic tissue was macroscopically normal, and subsequently confirmed as microscopically normal by histology. This cohort was examined exclusively by the FISH method outlined above. This sample cohort was not amenable to PCR methodologies.

#### Fresh colonic biopsy specimens

A total of 145 individuals were recruited for the prospective fresh biopsy study, 86 formed the ulcerative colitis (UC) cohort and were analysed alongside a cohort of 59 healthy controls (HC). Of the UC cohort 9 individuals were excluded, 3 could not undergo colonoscopy for clinical reasons and 6 had an alternative final diagnosis. The HC cohort comprised two groups. The first group (N = 32) were recruited specifically for this study and had biopsies collected for both FISH and PCR studies (as outlined above). The second group (N = 27) had been recruited previously and all 27 had biopsies collected from normal colon whilst undergoing polypectomy.

Biopsies were collected during colonoscopy using standard endoscopic forceps (Boston Scientific Nanterre Cedex France). The colonic mucosa was rinsed with sterile water via the colonoscope to remove residual faecal material. Biopsies were immediately snap frozen in liquid nitrogen and then transferred to a −80°C freezer until used for DNA analysis. Additional biopsies were also sent for histopathology assessment and FISH analysis.

Biopsies were only collected for PCR based studies and so FISH analysis was not studied in this cohort. Therefore mucosal biopsies were obtained from 136 individuals, 77 with a clinical and histological diagnosis of UC (55 established disease – three had antibiotic therapy in the 6 months prior to study recruitment, 22 *de-novo*) and 59 healthy controls. The entire UC group had biopsies collected for both fluorescent *in-situ* hybridisation (FISH) and PCR studies. Samples that generated positive PCR results with *Helicobacteraceae* or *H. pylori* PCR primers were sequenced to confirm identity. Based on sequence analysis results, samples that were suspected of containing multiple *Helicobacter* sequences were cloned [12] and 5 clones per sample were sent for sequence analysis (400 bp).

Ethical approval for the archival specimen analysis and the biopsy study was granted by North East of Scotland Research Ethics Service and written informed consent was obtained from all subjects in the biopsy study.

### Statistical analysis

Statistical analysis with the Pearson Chi Squared 2 tailed test, was performed using SPSS statistics software version 17.0.1 (December 1 2008).

## Results

### Validation of Molecular Methods (PCR/FISH) for the Detection of *Helicobacteraceae* Organisms

Fifty of the gastric biopsy samples demonstrated FISH positivity using both *Helicobacteraceae* and *H. pylori* specific probes along with the universal Eub338 probe. The same 50 samples tested positive by PCR for *Helicobacteraceae*, *H. pylori* specific and universal bacterial primer sets with a subset of results (N = 10) subjected to sequence analysis which confirmed the presence of *H. pylori* (sequence identities >99%). The molecular results were 100% concordant with the findings of clinical investigation indicating that the combined approach had a high diagnostic sensitivity and specificity.

The combined molecular approach was then applied to 100 infectious diarrhoea samples. All infectious diarrhoea samples showed positivity with the universal bacterial FISH probe (Eub338) and the universal bacterial PCR. One diarrhoea sample was positive for *Helicobacter* by FISH and both *Helicobacteraceae* and *H. pylori* specific PCR with sequencing confirming the presence of *H. pylori*. As non-*pylori Helicobacter* organisms have been isolated from diarrheal samples previously, we performed a series of spiking experiments to confirm that the diarrheal samples were not inhibiting PCR amplification. Ten samples which were negative for *Helicobacteraceae* by both FISH and PCR were spiked with *H. pylori* DNA (500 pg to 0.5 pg) prior to PCR amplification. All spiked samples yielded positive PCR and subsequent sequencing results (>99% sequence similarity, over 360 bp, to the spiked *H. pylori* 16S rDNA gene sequence) confirming that if present these organisms would have been detected.

### *Helicobacteraceae* Prevalence in Human Colonic Tissue

#### Archival study

The UC archival cohort (n = 57, 44% Male) had a median age of 40 (range 15–82) at the time of colonoscopy and were classified as extensive (40%), left sided (37%) and proctitis (23%) according to the Montreal criteria [22]. A total of 284 biopsies were analysed with 46% of subjects having biopsies from inflamed and un-involved mucosa available (E1 n = 8, E2 n = 9, E3 n = 9) and the remainder having biopsies only from inflamed mucosa. The HC archival cohort (n = 49, 35% Male) had a median age of 42 (range 14–80) at the time of colonoscopy. A total of 127 biopsies were processed in triplicate to assess the presence of *Helicobacteraceae* from the available pathology blocks of the right and left colon including rectum. Subjects were considered to be positive if appropriate fluorescent organisms were observed in at least 1 slide. All fluorescent *in-situ* hybridisations were assessed alongside *H. pylori* positive gastric biopsy reference slides and a selection of *Helicobacter* reference strains. All of the 106 subjects had bacteria detected with the universal bacterial probe. Of the 55 subjects positive for *Helicobacteraceae*, 29 were from the UC cohort and 26 were from the HC cohort. In both groups there was no statistically significant correlation with gender or age of the subjects and in the UC cohort the extent of disease did not correlate with the rate of positivity either as detailed in Table 3. Interestingly there was a statistically significant difference in the presence of *Helicobacter pylori* with 13 of the *Helicobacteraceae* positive subjects from the HC cohort also having a positive result for *Helicobacter pylori* compared with only 3 of the UC cohort (p = 0.002). Assuming that these *Helicobacter pylori* positive results represent transported gastric *Helicobacter pylori* we consequently removed these from the *Helicobacteraceae* positive results, thus creating a new category of non-pylori *Helicobacter* positive organisms. By this approach there was also a statistically significant difference in the presence of non-*pylori Helicobacter* organisms between the UC and HC cohorts (p = 0.04). There was no correlation with age, gender or severity of disease as detailed in Table 3. Because of limitations of the archival FISH technique it was not possible to ascertain if the *H. pylori* organisms were entirely responsible for the genus positivity or if the *H. pylori* were cohabiting with other non-*pylori Helicobacter*. Indeed, it was also not possible to ascertain if more than one *Helicobacteraceae* species was present within a sample. Attempts were made to extract microbial DNA from the archival tissue but these were unsuccessful and therefore a prospectively collected cohort was established with samples taken for PCR based analyses and FISH analysis.

**Table 3:** Archival Study FISH results.

	Gender	Montreal Extent of Disease	Median Age	Eub 338 +ve (%)	HFam +ve (%)	HP +ve (%)	NpH +ve (%)
**UC**	**Male**	1	29	8 (100)	3 (38)	0 (0)	3 (38)
		2	45	8 (100)	6 (75)	1 (13)	5 (63)
		3	40	9 (100)	6 (67)	1 (11)	5 (56)
		Total	32	25 (100)	15 (60)	2 (8)	13 (52)
	**Female**	1	37	5 (100)	3 (60)	0 (0)	3 (60)
		2	49	13 (100)	4 (31)	0 (0)	4 (31)
		3	39	14 (100)	7 (50)	1 (7)	6 (43)
		Total	42	32 (100)	14 (44)	1 (3)	13 (41)
	**Combined Total**		**40**	**57 (100)**	**29 (51)**	**3 (5)** *	**26 (46)** **
**Control**	**Male**		39	17 (100)	10 (59)	6 (35)	4 (24)
	**Female**		47	32 (100)	16 (50)	7 (22)	9 (28)
	**Combined Total**		**42**	**49 (100)**	**26 (53)**	**13 (27)** *	**13 (27)** **

*p = 0.002 (Pearson Chi Squared 2 tailed test).

**p = 0.04 (Pearson Chi Squared 2 tailed test).

#### Prospective study

The prospective UC cohort (n = 77, 46% Male) had a median age of 42 (range 16–84) at the time of index colonoscopy and were classified as extensive (25%), left sided (62%), proctitis (13.0%). A total of 137 biopsy sites were analysed. Twenty one (27%) subjects had a single site analysed, all of which represented inflamed mucosa and 56 (73%) had more than one biopsy site assessed, of which 42 both had inflamed and uninvolved mucosa. The prospective HC cohort (n = 59, 59% Male) had a median age of 63 (range 30–75) at the time of index colonoscopy. There was a statistically significant median age difference between the UC cohort and the control groups (Mann Whitney U test p<0.001). A single biopsy site (n = 59) was analysed from each control subject. All 136 subjects (77 UC and 59 controls) had colonic biopsies available for PCR analysis and all were positive for universal bacterial PCR indicating the presence of bacterial DNA within all samples. Of the 136 subjects, 43 (32%) were PCR positive for *Helicobacteraceae* and 3 (2%) were PCR positive for both *Helicobacter pylori* and *Helicobacter* genus (Table 4). Subsequent sequence analysis of the *Helicobacteraceae* PCR products confirmed the presence of only *Helicobacter pylori* in these 3 samples. Thus 40 subjects were PCR positive for *Helicobacteraceae* but not *H. pylori*. Sequence analysis revealed a further 4 subjects (3 controls and 1 UC) with only *Helicobacter pylori* identified.

**Table 4:** Prospective Study PCR results

	Gender	Montreal Extent of Disease	Median Age	Universal Bacteria +ve (%)	HFam +ve (%)	Gastric Species (%)	EHH species (%)
**UC**	**Male**	1	53	2 (100)	1 (50)	0 (0)	1 (50)
		2	44	21 (100)	10 (48)	1 (5)	9 (43)
		3	53	12 (100)	7 (58)	0 (0)	7 (58)
		Total	45	35 (100)	18 (51)	1 (3)	17 (49)
	**Female**	1	52	8 (100)	2 (25)	1 (13)	1 (13)
		2	42	27 (100)	9 (33)	0 (0)	9 (33)
		3	28	7 (100)	3 (43)	0 (0)	3 (43)
		Total	41	42 (100)	14 (33)	1 (2)	13 (31)
	**Combined Total**		**42**	**77 (100)**	**32 (42)** *	**2 (3)** **	**30 (39)** ***
**Control**	**Male**		61	35 (100)	6 (17)	4 (11)	2 (6)
	**Female**		64	24 (100)	5 (21)	5 (21)	0 (0)
	**Combined Total**		**63**	**59 (100)**	**11 (19)** *	**9 (15)** **	**2 (3)** ***

*p = 0.004 (Pearson Chi Squared 2 tailed test).

**p = 0.007 (Pearson Chi Squared 2 tailed test).

***p<0.0001 (Pearson Chi Squared 2 tailed test).

In the remaining 36 subjects sequence analysis identified the presence of a further 8 *Helicobacter* species and a *Wollinella succinogenes* with 6 subjects having more than one species identified (Table 5). Of these subjects, 3 had multiple *Helicobacter* species present within the same biopsy sample and 3 had different *Helicobacter* species spread between the samples analysed (Table S1). *Helicobacter pylori* were not found to co-exist with any other *Helicobacteraceae* species. The species identified and the number of samples they were identified in is detailed in Table S1.

**Table 5:** *Helicobacteraceae* species identified by sequencing.

*Helicobacteraceae* Species Identified	Number of Subjects Combination of Species identified in	
	UC	Control
**Single species identified**		
*Helicobacter cinaedi*	1	0
*Helicobacter canadensis*	1	0
*Helicobacter cholecystus*	9	0
*Helicobacter hepaticus*	5	1
*Helicobacter mustelae*	0	4
*Helicobacter pullorum*	5	1
*Helicobacter pylori*	2	5
*Wolinella succinogenes*	1	0
**Two species co-existing within Subject**		
*Helicobacter brantae* *Helicobacter pullorum*	1	0
*Helicobacter cholecystus* *Helicobacter bilis*	1	0
*Helicobacter cholecystus* *Helicobacter canadensis*	1	0
*Helicobacter cholecystus* *Helicobacter hepaticus*	3	0

*Helicobacteraceae* PCR positivity was significantly higher in UC than controls 32 of 77 (42%) versus 11 of 59 (19%), p = 0.004). By analysing the sequences obtained and including only those *Helicobacter* species classified as “enterohepatic,” the prevalence was 29 of 77 (38%) in the UC group versus 2 of 59 (3%) in the controls (p<0.0001). There was also a negative association between the identification of gastric *Helicobacter* species in UC (2 of 77) versus controls (9 of 59, p = 0.007) (Table 4). There was no correlation between the age, gender or extent of disease.

The effect of bowel preparation on *Helicobacteraceae* PCR positivity was also considered. Of the 77 UC subjects, 32 had full bowel preparation prior to colonoscopy along with all 59 control subjects. *Helicobacteraceae* PCR positivity in subjects with bowel preparation was significantly higher in UC than controls [20 of 32 (63%) versus 11 of 59 (19%), (Pearson Chi squared test p<0.0001)]. There was also a significant difference in *Helicobacteraceae* PCR positivity within the UC cohort based on bowel preparation 20 of 32 (63% full bowel preparation), 1 of 5 (20% phosphate enema preparation) vs 11 of 40 (28% no bowel preparation), Pearson Chi squared test p = 0.007). There was also no statistically significant association with antibiotic usage.

For 109 (77 UC, 32 HC) of the 136 subjects, samples were available for both FISH and PCR based analyses. All samples analysed by FISH were Eub338 positive indicating the presence of bacteria. 62 of 77 (81%) UC were *Helicobacteraceae* positive whilst 1 was also *H. pylori* positive. In the 32 controls, 12 (38%) were *Helicobacteraceae* positive whilst 2 were also *H. pylori* positive (Table 6). The *Helicobacteraceae* positivity was significantly higher in the UC cohort (p<0.0001) but no negative association with *Helicobacter pylori* as seen in the archival FISH and prospective PCR studies. As in the other studies there was no correlation between the age, gender or extent of disease. Correlation between PCR and FISH results for these 109 subjects were examined which demonstrated concordance in 74 (68%) of subjects. The majority of discordant results were attributable to a higher positivity rate for *Helicobacteraceae* with FISH than PCR (Table 7).

**Table 6:** Prospective Study FISH results.

	Gender	Montreal Extent of Disease	Median Age	Eub 338 +ve (%)	HFam +ve (%)	HP +ve (%)	NpH +ve (%)
**UC**	**Male**	1	53	2 (100)	2 (100)	0	2 (100)
		2	44	21 (100)	16 (76.2)	1 (4.8)	15 (71.4)
		3	53	12 (100)	12 (100)	0	12 (100)
		Total	45	35 (100)	30 (85.7)	1	29 (64.4)
	**Female**	1	53	8 (100)	8 (100)	0	8 (100)
		2	42	27 (100)	20 (74.1)	0	20 (74.1)
		3	28	7 (100)	4 (57.4)	0	4 (57.4)
		Total	41	42 (100)	32 (76.2)	0	32 (76.2)
	**Combined Total**		**42**	**77** (100)	**62 (85.7)** *	**1 (1.3)**	**61 (79.2)** **
**Control**	**Male**		64	15 (100)	5 (33.3)	0	5 (33.3)
	**Female**		64	17 (100)	7 (41.2)	2 (11.7)	5 (29.4)
	**Combined Total**		**64**	**32** (100)	**12 (37.5)** *	**2 (6.3)**	**10 (31.1)** **

*p<0.0001 (Pearson Chi Squared 2 tailed test).

**p<0.0001 (Pearson Chi Squared 2 tailed test).

**Table 7:** Correlation of PCR and FISH results.

	FISH *Helicobacter* negative (%)	FISH *Helicobacteraceae* positive (%)	FISH *H. pylori* positive (%)
**PCR ** ***Helicobacter*** ** Negative**	35 (32%)	35 (32%)	0
**PCR ** ***Helicobacteraceae*** ** positive**	0	39 (36%)	2 (2%)
**PCR ** ***H. pylori*** ** positive**	0	0	1 (1)

## Discussion

During the development of our combined molecular approach, both the PCR and FISH techniques were highly sensitive and specific (100% each) when interrogating gastric biopsies with known *H. pylori* status. A further validation cohort utilising diarrhoeal samples demonstrated limited *Helicobacteraceae* positivity. However spiking experiments on this cohort indicate that organisms would have been identified had they been present. These findings suggest that *Helicobacteraceae* are not a prominent causative agent in infectious diarrhoea in our setting although they have been isolated from diarrhoeal samples by other investigators [23]–[33]. Part of the rationale behind our developing a combined approach to identify *Helicobacteraceae*, rather than one based solely on either PCR or FISH, was that the combination of techniques allows visualisation of organisms *in-situ* and species-level identification from sequencing. PCR-only studies can be criticised based on the possibility that contaminant environmental DNA could bias results. In the gastrointestinal tract for instance, DNA could be transited to the colon in the faecal stream from foodstuffs. FISH addresses these concerns by allowing direct visualisation and localisation of organisms to the colonic mucosa. FISH-only studies however are limited by the constraints of designing species-specific probes and therefore they lack species-level sensitivity at times. By utilising both approaches *Helicobacteraceae* species present could be visualised and also identified. Based on the strength of these validation studies, we considered that this combined methodology was suitable for investigating *Helicobacteraceae* prevalence in UC colonic biopsies. A combined FISH/PCR approach has also been utilised to examine *Helicobacteraceae* prevalence in a small cohort of children with IBD (n = 12) (Crohn's disease n = 11), irritable bowel syndrome (IBS; n = 5) and controls (n = 4) [9]. This small study identified a strikingly high prevalence in both IBD (11/12) and IBS (5/5) versus controls (1/4). Through sequence analysis of DGGE bands several *Helicobacteraceae* were identified including *Helicobacter ganmani*, *Wolinella succinogenes*, *H. hepaticus* and *H. pylori*. Two further bands were identified as *Helicobacter* although equal sequence similarity was attributed to multiple species.

The results of our study show that rates of *Helicobacteraceae* positivity are significantly higher in the colonic tissue of UC patients than in controls. When sequencing data is analysed and species identities attributed, in control patients, the species identified are almost exclusively gastric (namely *H. pylori* and *H. mustelae* (99–100% sequence similarity); comprising 9 of 11). This finding is in stark contrast to the *Helicobacter* sequences from the UC cohort where 32 patients had *Helicobacteraceae* species identified, although only 2 of these were attributed to *H. pylori* and curiously, *H. mustelae* was absent. Our study was not designed to obtain gastric *Helicobacter* species. As such it was not possible to confirm or refute the notion that *Helicobacter* species detected in the colon are truly colonising the mucus or merely transiting from the stomach. For the former, *Helicobacter* grown from both sites (stomach and colon) would be necessary in order to undertake a detailed strain comparison, which was not feasible within this study. As indicated in the results section there was also a statistically significant difference in age between the UC and control groups. The difference in age between the two groups is a result of the control group predominantly being recruited from a colorectal cancer screening programme whose lower age limit is 50 years.

Interestingly, bowel preparation appeared to increase the detection of *Helicobacter* species. It might be anticipated that the wash-out effect of bowel preparation might reduce the positivity. This surprising result may be the result of an unidentified confounding factor or could be a surrogate disease severity marker. This was due to it being a clinical decision whether subjects received bowel preparation or not. As no bowel preparation was given when there was the clinical impression of severe disease. This suggests that *Helicobacteraceae* positivity is associated with less severe disease. However no association between *Helicobacteraceae* positivity and the Montreal classification of disease extent and severity was observed.

Six of the UC patients appear to have multiple *Helicobacter* species present within their colonic tissue. Two of the six had different *Helicobacter* sequences identified from the same biopsy whereas the other four had single *Helicobacter* species identified from biopsies taken from different regions of the colon. Mixed species were not identified in the control cohort although it should be acknowledged that only single colonic sites were investigated by biopsy. Nevertheless our findings suggest that more than one enterohepatic *Helicobacter* species can be present in the same human host although this does not appear to be the case for gastric species.

It should be noted that allocation of species should not rely solely on 16S rRNA sequencing as comparison of these sequences can be misleading and does not always provide conclusive evidence for species level identification. *Helicobacter* species identity cannot be firmly established by 16S sequencing and the 400 bp product of the nested PCR further compounded this by only allowing sequencing over this short segment. However there is confidence that the sequence belonged to the genus *Helicobacter* based on the sequenced product. For example, although our nested PCR technique amplified a hyper-variable region of the 16S rRNA *Helicobacter* genome which equated to an estimated average evolutionary diversity of 14 base pairs within the 9 species identified [34], it is not possible to be certain of the *Helicobacter* species without additional genotypic or phenotypic characterisation [35]. There is always the possibility that mixed *Helicobacter* organisms were present that had identical sequences over the 400 bp 16S rDNA region analysed. It is possible that alternative identification approaches including denaturing gradient gel electrophoresis (DGGE) could have been used to address this potential issue. Where sequence analysis indicated that mixed species were present, the additional cloning and sequencing approach was undertaken which demonstrated the presence of multiple *Helicobacter* sequences within a few samples.

The use of nested PCR is known to increase the sensitivity of the PCR test; however it is also known that without strict use of appropriate control strategies including sequencing of positive results, it can lead to false positives. In the current study, all positive nested PCR findings were sequenced in order to eliminate the query of false positives due to nested PCR. Extensive attempts were made to isolate *Helicobacter* species from the prospective cohort, however this was not successful. *Helicobacter* species are notoriously fastidious and although novel *Helicobacter* species have been isolated from human faeces, to date none have been isolated from colonic tissue. Examining both FISH and PCR analyses on a large number of samples (n = 109) revealed a correlation rate of ∼68%. In the majority of cases, discordant results showed that FISH was more likely to yield a positive result than PCR. This was not seen in our gastric validation cohort. The most likely explanation is that the number of *H. pylori* strains present in infected gastric tissue is higher than the corresponding number of *Helicobacter* species in the colon. These results suggest that the FISH technique is more sensitive but lacks the specificity to identify the *Helicobacteraceae* species. Regardless of these technical issues, both techniques demonstrate a statistically significant correlation between the presence of *Helicobacteraceae* species, particularly the enterohepatic *Helicobacter* species and the UC cohort.

The PCR methodology that we developed used a nested PCR approach for *Helicobacteraceae* but a single PCR was used for *H. pylori*. It is likely that a nested PCR for *H. pylori* would have been more sensitive in the colonic samples and would have increased the detection rate. This is a plausible explanation for at least 3 of the four samples which were shown by sequence analysis of the *Helicobacteraceae* PCR product to contain *H. pylori* despite *H. pylori* PCR results being negative. Nevertheless, in order to identify every *Helicobacter* that was detected, we chose to sequence every positive PCR reaction including those that were *H. pylori* positive in order to determine whether multiple species were present.

Attributing causation to putative pathogens has always been a difficult endeavour with the gold standard remaining fulfilment of Koch's postulates [36]. In an era of molecular biology and an increasing awareness of the “unculturable” microbiota of the human colon however, these postulates are perhaps outdated. Swidsinski and colleagues recently proposed alternative postulates for a modern era [37]:

- There must be a clear link between a pathogen and a disease,
- The pathogenic organism should be identified and characterised (by traditional culture and phenotyping or by “reliable” modern methods such as PCR, DNA sequencing and FISH),
- There should be positive evidence of the chain of infection (this can be from individual transfections or from epidemiological observation)
- Knowledge of a specific pathogen should assist the development of new diagnostic methods and treatment

We would add that host factors, in particular genetic or immunological susceptibility should be considered, particularly in the context of IBD. We believe that our data adds considerable weight to fulfilling Swidsinski's second postulate and that the first has already been firmly established in animal models. Further work is required to address the third postulate which would clearly be aided by successful culture of these organisms from the colonic tissue of UC patients.

Finally, the presence of *H. mustelae* in the colonic tissue of controls but not UC patients warrants further consideration. Since *H. mustelae* is a gastric organism (previously only identified in ferrets [38]), it would be interesting to see if this species is co-colonising the human stomach and colon, the colon alone or simply being transited to the colon from the stomach. *H. mustelae* has a similar morphology to *H. pylori* and is also a urease positive organism so it could easily be mistaken with current clinical testing (CLO test) for *H. pylori* in human gastric disease. It may be that *H. mustelae* represents a less pathogenic organism in the human host which nonetheless confers the IBD protective benefits of *H. pylori* [39]. In terms of the extra-gastric *Helicobacters* our findings clearly demonstrate compelling molecular evidence for their presence in the human colon. Their presence in the human host is not well established compared to animal models however; there is no reason to suggest that they do not reside in the human intestinal tract. Clearly these hypotheses require further exploration.

## Supporting Information

Table S1 Bacterial Sequence Identification* of Helicobacteraceae positive samples.
(DOC)
