# Supplementary material for: Enterohepatic Helicobacter in Ulcerative Colitis: Potential Pathogenic Entities?
Source: PLoS One. 2011 Feb 23;6(2):e17184. doi: 10.1371/journal.pone.0017184 (PMC3044171; doi:10.1371/journal.pone.0017184)
Supplement: Table S1 — Bacterial Sequence Identification* of Helicobacteraceae positive samples. (DOC) [file pone.0017184.s001.doc]

**Table S1: Bacterial Sequence Identification* of *Helicobacteraceae*** positive samples

| **Subject** | **Age** | **Gender** | **Montreal Classification** | **Number of Samples Analysed**  **(PCR positive)** | **Mucosal State** | **BLAST Sequencing Hit(s)** |
| --- | --- | --- | --- | --- | --- | --- |
| 2UC1 | 34 | M | E1, S2 | 2 (1) | Inflamed | AM998803 *Helicobacter canadensis* CCUG 47163T (99%) |
| 2UC2 | 70 | F | E2, S2 | 2 (1) | Uninvolved | AB275317 *Helicobacter cinaedi* CCUG18818 (98%) |
| 2UC4 | 22 | M | E2, S1 | 3 (1) | Uninvolved | U07575 *Helicobacter hepaticus* strain Hh-3 (99%) |
| 2UC5 | 21 | F | E2, S2 | 3 (1) | Uninvolved | FJ236465 *Helicobacter pullorum* NCTC 12824 (100%) |
| 2UC6 | 60 | M | E2, S2 | 2 (1) | Inflamed | U07575 *Helicobacter hepaticus* strain Hh-3 (98%) AY686606 *Helicobacter cholecystus* ATCC 700242 (98%) |
| 2UC7 | 43 | F | E3, S0 | 2 (1) | Remission | AY686606 *Helicobacter cholecystus* ATCC 700242 (99%) |
| 1UC8 | 35 | F | E2, S3 | 2 (1) | Inflamed | M88159 *Wolinella succinogenes* ATCC 29543 (99%) |
| 2UC8 | 78 | M | E3, S1 | 2 (1) | Inflamed | FJ236465 *Helicobacter pullorum* NCTC 12824 (99%) |
| 2UC11 | 44 | M | E2, S2 | 2 (1) | Uninvolved | FJ236465 *Helicobacter pullorum* NCTC 12824 (99%) |
| 2UC12 | 60 | M | E3, S3 | 2 (1) | Inflamed | FJ236465 *Helicobacter pullorum* NCTC 12824 (100%) |
| 2UC14 | 52 | F | E2, S2 | 2 (1) | Inflamed | U07575 *Helicobacter hepaticus* strain Hh-3 (100%) |
| 2UC15 | 74 | F | E1, S1 | 2 (1) | Remission | CP001680 *Helicobacter pylori* (99%) |
| 2UC18 | 27 | M | E2, S2 | 2 (1) | Inflamed | U07575 *Helicobacter hepaticus* strain Hh-3 (98%) |
| 1UC19 | 29 | M | E2, S2 | 1 (1) | Inflamed | U07575 *Helicobacter hepaticus* strain Hh-3 (99%) |
| 2UC20 | 48 | M | E2, S1 | 2 (1) | Uninvolved | U07575 *Helicobacter hepaticus* strain Hh-3 (99%) |
| 2UC23 | 52 | M | E3, S0 | 2 (2) | Inflamed | U07575 *Helicobacter hepaticus* strain Hh-3 (99%) |
|  |  |  |  |  | Uninvolved | U07575 *Helicobacter hepaticus* strain Hh-3 (100%) |
| 2UC24 | 31 | F | E2, S2 | 2 (1) | Inflamed | AY686606 *Helicobacter cholecystus* ATCC 700242 (98%) |
| 2UC25 | 46 | M | E2, S2 | 2 (1) | Inflamed | CP001680 *Helicobacter pylori* (99%) |
| 2UC26 | 73 | F | E1, S1 | 2 (2) | Inflamed | AY686606 *Helicobacter cholecystus* ATCC 700242 (99%) |
|  |  |  |  |  | Uninvolved | AY686606 *Helicobacter cholecystus* ATCC 700242 (99%) |
| 2UC27 | 57 | M | E3, S2 | 2 (2) | Inflamed | AM998803 *Helicobacter canadensis* CCUG 47163T (99%) |
|  |  |  |  |  | Uninvolved | AY686606 *Helicobacter cholecystus* ATCC 700242 (99%) |
| 2UC28 | 41 | F | E2, S1 | 2 (1) | Inflamed | U07575 *Helicobacter hepaticus* strain Hh-3 (99%) |
| 2UC29 | 41 | F | E2, S1 | 2 (2) | Inflamed | U07575 *Helicobacter hepaticus* strain Hh-3 (100%) |
|  |  |  |  |  | Inflamed | AY686606 *Helicobacter cholecystus* ATCC 700242 (99%) |
| 2UC30 | 29 | M | E3, S0 | 1 (1) | Remission | AY686606 *Helicobacter cholecystus* ATCC 700242 (99%) |
| 2UC31 | 52 | M | E3, S0 | 2 (2) | Inflamed | AY686606 *Helicobacter cholecystus* ATCC 700242 (98%) |
|  |  |  |  |  | Uninvolved | AY686606 *Helicobacter cholecystus* ATCC 700242 (100%) |
| 2UC32 | 62 | M | E2, S2 | 3 (3) | Inflamed | AY686606 *Helicobacter cholecystus* ATCC 700242 (99%) |
|  |  |  |  |  | Uninvolved | AY686606 *Helicobacter cholecystus* ATCC 700242 (99%) |
|  |  |  |  |  | Uninvolved | AY686606 *Helicobacter cholecystus* ATCC 700242 (100%) |
| 2UC33 | 33 | F | E2, S2 | 2 (2) | Inflamed | FJ236465 *Helicobacter pullorum* NCTC 12824 (99%) |
|  |  |  |  |  | Uninvolved | DQ415546 *Helicobacter brantae* strain MIT 04-9366 (99%) |
| 2UC34 | 24 | F | E3, S2 | 2 (2) | Inflamed | AY686606 *Helicobacter cholecystus* ATCC 700242 (100%) |
|  |  |  |  |  | Inflamed | AY686606 *Helicobacter cholecystus* ATCC 700242 (99%) |
| 2UC35 | 61 | F | E2, S0 | 2 (2) | Remission | U18766 *Helicobacter bilis* strain Hb1 (100%) |
|  |  |  |  |  | Remission | AY686606 *Helicobacter cholecystus* ATCC 700242 (100%) |
| 2UC36 | 75 | M | E2, S2 | 2 (1) | Uninvolved | AY686606 *Helicobacter cholecystus* ATCC 700242 (99%) |
| 2UC38 | 25 | F | E3, S2 | 2 (2) | Inflamed | AY686606 *Helicobacter cholecystus* ATCC 700242 (100%) |
|  |  |  |  |  | Uninvolved | AY686606 *Helicobacter cholecystus* ATCC 700242 (100%) |
| 1UC35 | 72 | M | E3, S2 | 2 (1) | Inflamed | U07575 *Helicobacter hepaticus* strain Hh-3 (98%) AY686606 *Helicobacter cholecystus* ATCC 700242 (98%) |
| 2UC44 | 39 | M | E2, S1 | 2 (1) | Inflamed | AJ876520 *Helicobacter pullorum* ATCC 51863 (100%) |
| HC38 | 52 | M | NA | 1 (1) | Normal | FJ236465 *Helicobacter pullorum* NCTC 12824 (99%) |
| HC53 | 50 | M | NA | 1 (1) | Normal | CP001680 *Helicobacter pylori* (100%) |
| HC88 | 64 | F | NA | 1 (1) | Normal | M35048 *Helicobacter mustelae* (99%) |
| HC89 | 73 | M | NA | 1 (1) | Normal | M35048 *Helicobacter mustelae* (100%) |
| HC90 | 67 | F | NA | 1 (1) | Normal | M35048 *Helicobacter mustelae* (100%) |
| HC91 | 50 | M | NA | 1 (1) | Normal | M35048 *Helicobacter mustelae* (100%) |
| HC93 | 73 | F | NA | 1 (1) | Normal | CP001680 *Helicobacter pylori* (100%) |
| HC101 | 50 | M | NA | 1 (1) | Normal | U07575 *Helicobacter hepaticus* strain Hh-3 (99%) |
| HC103 | 68 | F | NA | 1 (1) | Normal | CP001680 *Helicobacter pylori* (100%) |

* Based on sequence analysis of 400bp PCR product
